# Addressing Cardiovascular Disparities in Racial/Ethnic Populations: The Blood Pressure-Lowering Effects of SGLT2 Inhibitors

**DOI:** 10.31083/j.rcm2312411

**Published:** 2022-12-19

**Authors:** Samar A. Nasser, Neha Arora, Keith C. Ferdinand

**Affiliations:** ^1^Department of Clinical Research & Leadership, School of Medicine and Health Sciences, The George Washington University, Washington, DC 20052, USA; ^2^Department of Medicine, Tulane University School of Medicine, New Orleans, LA 70112, USA

**Keywords:** SGLT2 inhibitors, hypertension, race/ethnicity, non-Hispanic Black patients, disparities, social determinants, blood pressure

## Abstract

The racial/ethnic disparities in cardiometabolic risk factors and cardiovascular diseases (CVD) are prominent in non-Hispanic Black adults and other United States (U.S.) sub-populations, with evidence of differential access and quality of health care. High blood pressure (BP) is the most potent and prevalent risk factor for adverse cardiovascular (CV) outcomes across all populations globally, but especially in the non-Hispanic Black adults in the U.S. The use of sodium-glucose cotransporter-2 inhibitors (SGLT2is) demonstrate favorable effects in patients with and without type 2 diabetes (T2DM) in CVD especially for heart failure (HF), as the contemporary clinical practice recommendations and standards of care advocate. The beneficial effects of SGLT2is have been most profoundly documented with HF, including reduced (HFrEF) or preserved ejection fraction (HFpEF), and chronic kidney disease (CKD) with T2DM. Given that hypertension (HTN), CVD, HF, and CKD are significantly greater in certain racial/ethnic populations, the potential impact of SGLT2is will be more significant on the excess cardiometabolic and renal disease, especially in the Black patients. Moreover, there is a need for increased diverse representation in clinical trials. Inclusion of larger members of various racial/ethnic populations may assure that new and emerging data accurately reflect the diversity of the U.S. population. This review highlights potential benefits of SGLT2is, as noted in the most recent literature, and their BP-lowering impact on potentially reducing CV disparities, especially in Black adults. Furthermore, this commentary emphasizes the need to increase diversity in clinical trials to reduce the disparity gaps.

## 1. Introduction

The racial/ethnic disparities in cardiometabolic risk factors and cardiovascular diseases (CVD) are prominent in non-Hispanic Black adults and other United States (U.S.) sub-populations, with evidence of differential access and quality of health care. High blood pressure (BP) is the most powerful and dominant risk factor for adverse cardiovascular (CV) outcomes across all populations globally, but especially in the non-Hispanic Black adults in the U.S. [1]. The use of sodium-glucose cotransporter-2 inhibitors (SGLT2is) demonstrate favorable effects in patients with and without type 2 diabetes (T2DM) in CVD especially for heart failure (HF), as the contemporary clinical practice recommendations and standards of care advocate. Worldwide, the number one cause of death is CVD which often coincides with diabetes, diabetic kidney disease (DKD) and other forms of chronic kidney disease (CKD), with CVD coupled to the development of DKD [2].

The beneficial effects of SGLT2is have been most profoundly documented with HF, including reduced (HFrEF) or preserved ejection fraction (HFpEF), and CKD with T2DM. Given that hypertension (HTN), CVD, HF, and CKD are significantly greater in certain racial/ethnic populations, the potential impact of SGLT2is will be more significant on the excess cardiometabolic and renal disease, especially in the Black patients. Moreover, there is a need for increased diverse representation in clinical trials. Inclusion of larger members of various racial/ethnic populations may assure that new and emerging data accurately reflect the diversity of the U.S. population. This review highlights potential benefits of SGLT2is, as noted in the most recent literature, and their BP-lowering impact on potentially reducing CV disparities, especially in Black adults. Furthermore, this commentary emphasizes the importance of diversifying clinical trials to reduce the disparity gaps.

## 2. Hypertension and Associated Cardiovascular Disease Burden

Hypertension is the most potent and prevalent CV risk factor in the U.S. and globally [3], and a persistent and increasing public health crisis. Notably, the prevalence of HTN (BP ≥130/80 mm Hg) among adults in the U.S. is approximately 45%, and even higher in non-Hispanic Black adults whose prevalence is among the greatest globally [3]. According to the pooled NHANES (National Health and Nutrition Examination Survey) 2011 to 2016, and individual-level data from seven U.S. community-based cohort studies, an estimated 70% of major CVD events in the U.S. were attributable to low and moderate CV health, with a preventable 2 million major CVD events yearly if all U.S. adults attain high CV health [4]. From 2017–2018, the U.S. incurred an estimated $378 billion annual direct and indirect cost of CVD [4].

Likewise, CVD is the primary cause of death in patients with diabetes, and control of associated risk factors leads to substantial reductions in CV events [1, 4]. From 1990 to 2019, T2DM increased from the ninth to the third leading cause of years of life with disability or injury rates, demonstrating a 55.8% increase [4]. Moreover, overweight and obesity status increases the risk of HTN, dyslipidemia, and T2DM. A report from the Framingham Offspring Study demonstrated higher risks of HTN, diabetes, CKD, CVD, and mortality associated with having a shorter duration of ideal CV health in adulthood [5].

The racial/ethnic disparities in cardiometabolic health are prominent in Black adults with: more severe and prevalent HTN, increased T2DM, more extensive and severe obesity (especially in Black women), premature myocardial infarction (MI), increased stroke and related mortality, more prevalent CKD, higher risk for end stage renal disease, HF-related death, and premature CV mortality [3, 6]. The differential access to quality health care is omnipresent, falling on the backdrop of historic and current social and economic practices and policies that lead to inequalities across many U.S. healthcare systems [7]. Presently, the public health crisis is more recognized secondary to the confluence of national concerns regarding social injustice, racial inequities, and enduring health disparities.

**Fig. 1.**
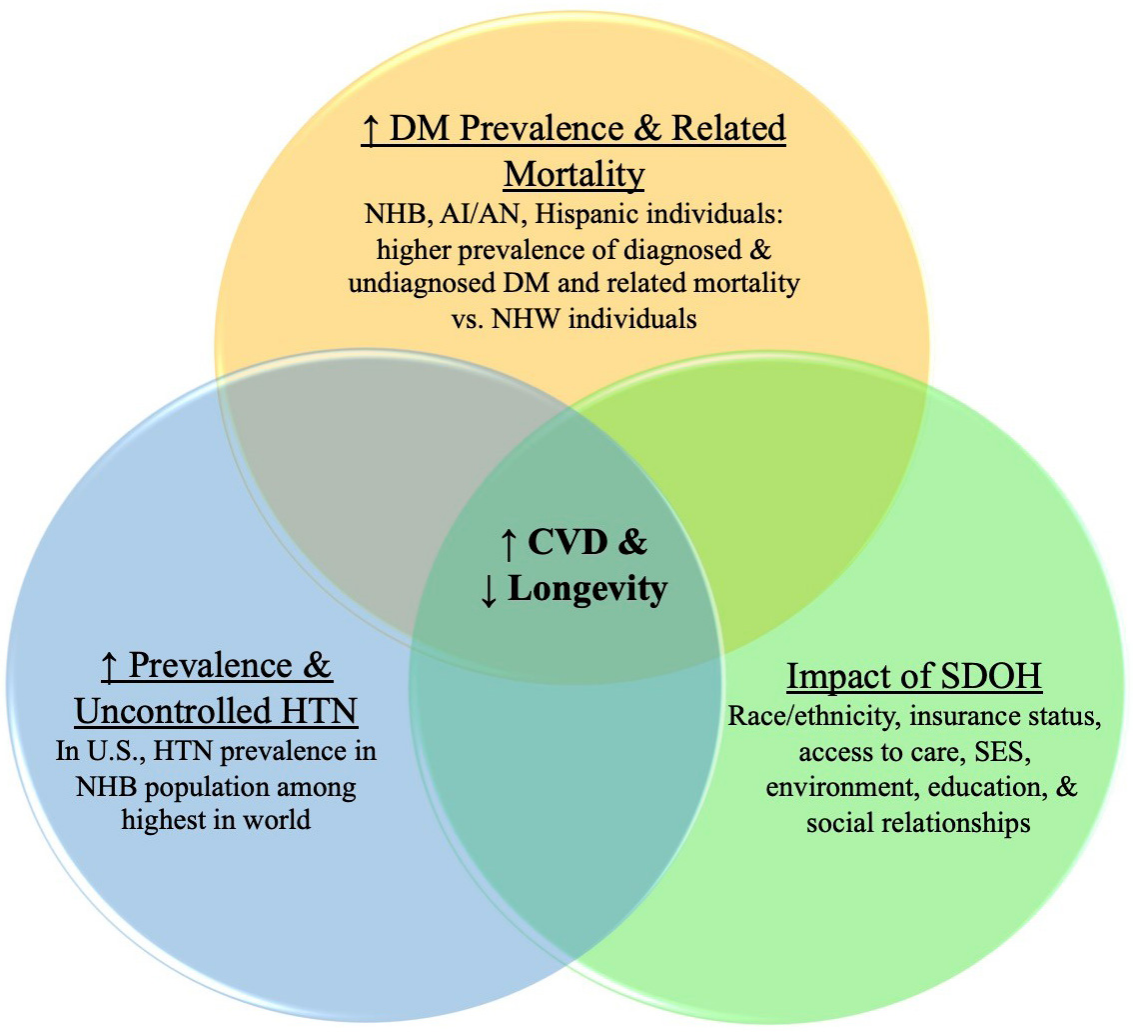
**Predominant factors affecting cardiometabolic/CV burden and longevity in racial/ethnic populations**. The combination and amplification of multiple negative factors in racial/ethnic populations increases CVD and decreases longevity. NHB, non-Hispanic Black; AI/AN, American Indian/Alaska Native; NHW, non-Hispanic White; DM, Diabetes; HTN, Hypertension; SDOH, Social determinants of health; SES, Socioeconomic status.

Recently, Ouyang and colleagues [8] investigated the mortality trends of non-communicable diseases, including comorbid HTN and the disparities in mortality rates across various demographic subgroups. In 2019, HTN-related CVD mortality age-standardized rates in the Black population was 1.46 times higher than in the White population (62.5 per 100,000 *vs*. 42.7 per 100,000) among both men and women. Accordingly, the Black–White morbidity and mortality gaps are primarily explained by the advanced disease stages at diagnosis and delayed treatment among the Black population [8]. Moreover, the social determinants of health (SDOH) reflect the non-medical factors that influence health outcomes, including socioeconomic status, poverty, and access to primary care, and contribute to the overwhelming health disparities [9]. For instance, the practice of redlining in the 1930’s, in which largely Black and immigrant neighborhoods were deemed “hazardous”, created geographical spaces of individuals who now have markedly decreased healthcare and insurance coverage, public transport access, and healthy food choices [10]. Current residents of these historically redlined neighborhoods thus experience higher rates of cardiometabolic disease, including diabetes, obesity, and coronary artery disease, compared to those in green neighborhoods (Fig. 1).

## 3. Unacceptable Cardiovascular Mortality Gap among Racial/Ethnic Populations

The racial/ethnic disparity mortality gaps are primarily driven by CVD and cardiometabolic conditions [7]. In recent years, life expectancy in the U.S. has stagnated, and then slowly declined [11]. According to Woolf and colleagues, the U.S. life expectancy decreased from 78.86 years in 2019 to 76.60 years in 2021, a resulting in a decrease of 2.26 years [12]. Even more significant and reflecting racial disparities in the U.S., the life expectancy demonstrated greater decreases in the Hispanic (3.70 years; credible range (CR), 3.53–3.87 years) and Black (3.22 years; CR, 3.03–3.40 years) populations than in the non-Hispanic White population (1.38 years; CR, 1.21–1.54 years) [12]. Over the past few decades, there has been a consistent mortality gap between non-Hispanic White and Black adults. Overall, there is an increase in the aging of the population, decrease in physical activity, and increase in prevalence of obesity, HTN, and diabetes. The culmination of these chronic conditions will result in a tsunami with an enormous wave of death and disability that demands immediate multidisciplinary and comprehensive strategies to address.

## 4. Mechanisms of SGLT2 Inhibitors in Cardiometabolic and Cardiovascular Disease

The use of the glucose-lowering agents, SGLT2is, has recently demonstrated advantageous effects of reducing the onset and progression of renal complications, regardless of blood sugar control [13]. The key mechanism of action of SGLT2is target the luminal side of the proximal tubules, as SGLT2is accelerate urinary glucose excretion by inhibition of glucose reabsorption in the renal proximal tubules. Additionally, SGLT2is lead to diuretic effects by inhibiting the absorption of sodium with the glucose from the proximal tubule, resulting in increased urinary sodium loss. Moreover, SGLT2is improve tubular oxygenation and metabolism and decrease renal inflammation and fibrosis [13].

There are also data demonstrating a reduction in adverse CVD outcomes, including HF [14]. Accordingly, patients receiving SGLT2is showed a comparable risk for MI/stroke/mortality, but a lower risk for hospitalization for HF/mortality and hospitalization for HF when compared with those receiving metformin [14]. Overall, the CVD benefits of SGLT2is are not solely dependent upon glycemic control and may also be considered in those with T2DM and CVD, independent of their hemoglobin A1c goal attainment [15, 16]. Thus, treatment with SGLT2is significantly decreases the rate of CV events (especially HF) and prevents the progression of renal dysfunction in patients, with or without diabetes, who were already receiving optimal guideline-directed treatment [17].

**Fig. 2.**
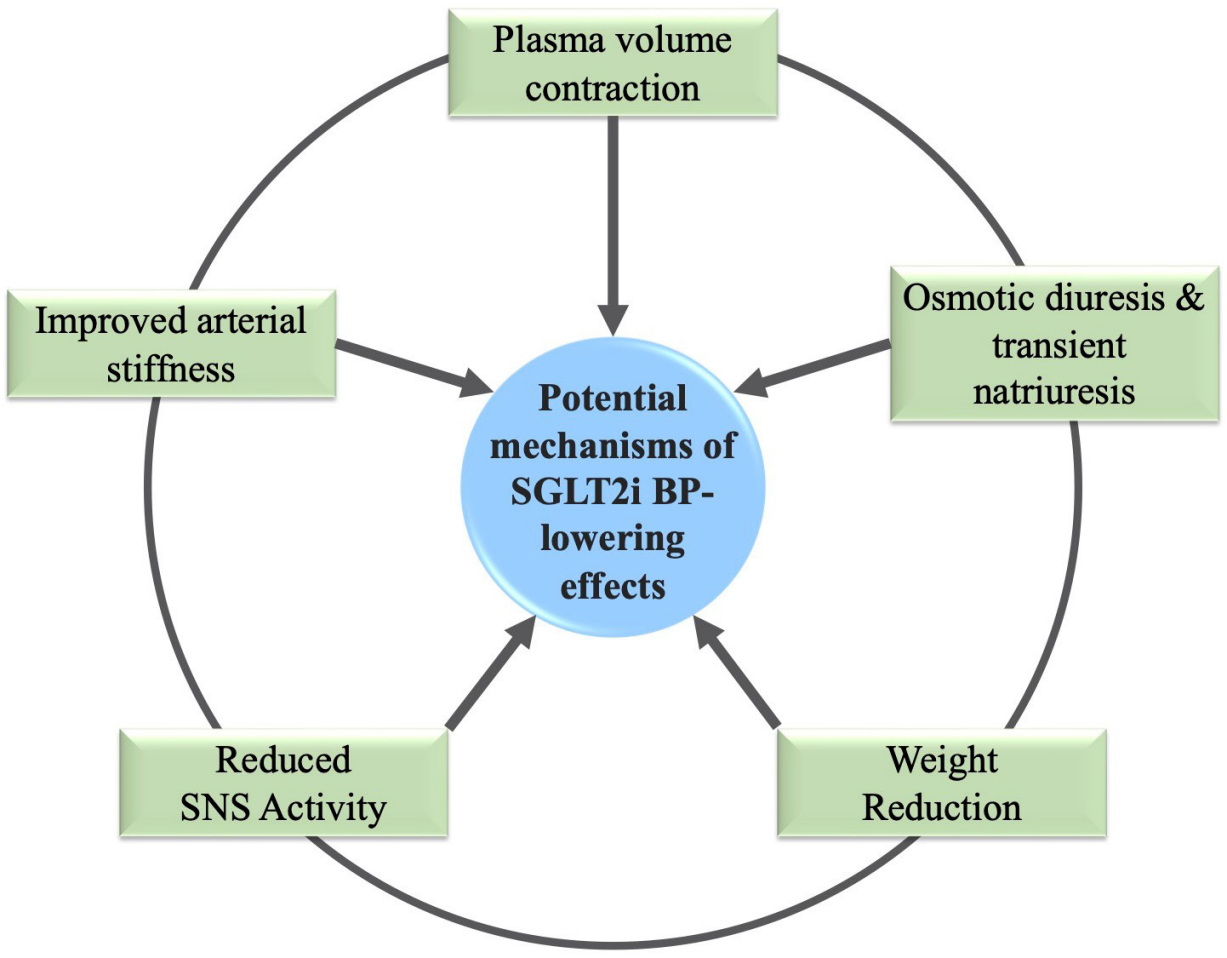
**Blood Pressure-Lowering Effects of SGLT2is**. SGLT2is impact many different physiologic processes that potentially contribute to their BP-lowering effects. BP, Blood pressure; SNS, Sympathetic nervous system.

Of note, these effects have also been shown in non-diabetic, lean and normotensive individuals; however, additional mechanisms are likely to contribute to the BP-lowering effects. SGLT2is reduce BP without increasing heart rate, which may suggest dampening of sympathetic nervous system activity [18]. Overall, SGLT2is support mechanisms reaching beyond glucose, weight, and BP-lowering effects that accompany their glucosuric action in patients with diabetes. Furthermore, SGLT2is are generally well tolerated, despite a potential risk for genital mycotic infections, but have not been evaluated in those with severe renal impairment (estimated glomerular filtration rate <25 mL/min/1.73 $m^{2}$ [19]. The proposed mechanisms of SGLT2is are summarized in Fig. 2.

## 5. The Potential Role of SGLT2 inhibitors in Hypertension

In consideration of the overarching contribution of elevated BP to CVD, the antihypertensive effects of SGLT2is may be a significant contributor to reducing racial/ethnic CV health disparities. In order to demonstrate the BP-lowering effects in a high-risk Black population, Ferdinand and colleagues demonstrated that SGLT2is reduce 24-hour mean systolic blood pressure (SBP) by 8.5 mm Hg (placebo-corrected) over the course of 24 weeks. The confidence interval indicated an effect as large as 13.7 mm Hg and a minimum effect of 3 mm Hg [20]. Additionally, among those patients with Stage 2 HTN, there was a more robust drop in BP as the mean BP was greater (mean decrease of 10.33 versus placebo).

Furthermore, in Phase III studies, Kario and colleagues observed BP reductions on average of 5.5 mm Hg in SBP and 1.5 mm Hg in diastolic blood pressure (DBP) [17]. Additional studies demonstrated similar reductions of SBP by 2.5 mm Hg and DBP by 1.5 mm Hg, and an average reduction of 24-hour ambulatory SBP and DBP by 3.8 mm Hg and 1.8 mm Hg, respectively [17, 21]. Moreover, the results were consistent across different SGLT2is in comparison with placebo or with other hypoglycemic medications in those already taking antihypertensive [17, 21]. Notably, the reduction in BP seems to be independent from improved glycemic control.

In a post hoc analysis of the CREDENCE (Canagliflozin and Renal Events in Diabetes with Established Nephropathy Clinical Evaluation) trial, investigators assessed the effect of canagliflozin on SBP across subgroups and whether effects on clinical outcomes differed across these subgroups [22]. CREDENCE trial randomized 4401 participants with T2DM and CKD to canagliflozin or placebo, of whom 3361 (76.4%) had baseline SBP ≥130 mm Hg, and 1371 (31.2%) had resistant HTN. Canagliflozin reduced SBP by 3.50 mm Hg (95% CI, –4.27 to –2.72) by the third week which was sustained throughout the trial. Thus, SGLT2i, canagliflozin, demonstrated early and sustained reductions in SBP in patients with T2DM and CKD, regardless of baseline BP [22].

According to the 2022 American College of Cardiology/American Heart Association/Heart Failure Society of America guideline, SGLT2is are recommended in patients with and without T2DM, specifically to prevent hospitalizations and associated mortality for HF [23]. Moreover, the American Diabetes Association recommends the use of SGLT2is as first-line agents for the treatment of hyperglycemia in patients with diabetes with HF or at high risk of HF [24]. SGLT2is are the first class of glucose-lowering agents to receive approval from the Food and Drug Administration (FDA) for the treatment of HF and reduced EF. Importantly, SGLT-2is are crucial because improved glycemia does not account for the CV benefit [25]. Recently, a retrospective cohort analysis using mortality data of patients aged 15 to 44 years demonstrated HF-related age-adjusted mortality rates increased for all racial/ethnic groups, with Black adults having the greatest (6.41 in 1999 and 8.58 in 2019) [26]. Comparatively, Hispanic and White adults increased from 1.62 to 2.04 and 1.83 to 2.45 over the same time period, respectively. Considering the younger mortality of HF among Black patients, the use of SGLT2is to address the rising burden of HF in young adults may be critical to reduce the gap in disparities. A recent meta-analysis demonstrated that SGLT2is are an effective class of drugs for improving CV morbidity and mortality in specific patients, including prevention of HF hospitalization [27]. To evaluate the association between SGLT2is and CV benefits, Bhattarai and colleagues [27] obtained data from ten randomized clinical trials with 71,553 participants supporting SGLT2is as an effective class for improving CV morbidity and mortality in selected patients.

## 6. Future Research with SGLT2 Inhibitors and the Need for Diversity in Clinical Trials

Diverse representation in clinical trials is vital for clinicians and researchers to be assured that the data accurately reflect the heterogenous U.S. population. New therapies can be especially applicable for disparate populations, but clinicians and researchers must ensure these treatments are as effective and safe for Black and other racial/ethnic populations. Therefore, multifaceted efforts that address these barriers are needed to recruit patients and prevent cardiometabolic complications, specifically in racial/ethnic populations.

Overall, HTN, CVD, HF, and CKD co-morbidities each and collectively are more significant in racial/ethnic populations, and thus the potential benefits of SGLT2is may have a greater impact on cardio-renal disease in the Black population. Unfortunately, there are limited numbers of racial/ethnic participants in clinical trials. Accordingly, Hoppe and colleagues reviewed data from seven CV outcome trials from the 2008 FDA mandate for evaluating CV risk for new therapies in T2DM (Table 1, Ref. [28, 29, 30, 31, 32, 33, 34, 35]).

**Table 1.** **Minority representation in major cardiovascular outcome trials in type 2 diabetes**.

	N	Drug (*vs* placebo)	Percentage total	Number and percentage
			black or African American	US or North American participants
EXAMINE (NCT00968708; Takeda) [29]	5380	Alogliptin	4.0%	744/5380 (13.9%)
EMPA‐REG OUTCOME (NCT01131676) [30]	7064	Empagliflozin	5.1%	1394/7064 (19.7%) (participants also include Australia and New Zealand)
SAVOR‐TIMI 53 (NCT01107886) [31]	16,492	Saxagliptin	3.4%	5266/16492 (31.9%)
ELIXA (NCT01147250) [32]	6068	Lixisenatide	3.6%	696/6068 (11.5%)
TECOS (NCT00790205) [33]	14,671	Sitagliptin	3.0%	2045/14671 (13.9%)
LEADER (NCT01179048) [34]	9340	Liraglutide	8.3%	2847/9340 (30%)
SUSTAIN‐6 (NCT01720446) [35]	3297	Semaglutide	6.7%	1137/3297 (34.5%)

Table adapted from supplement: Hoppe C, Kerr D. Minority underrepresentation in cardiovascular outcome trials for type 2 diabetes. Lancet Diabetes Endocrinol 2017; 5: 13. [28].

Overall, Black participation in these massive trials was less than 5% in five of the seven trials [28]. Thus, clinicians may have less confidence in the outcomes, and dissemination to the population is less robust. Recent data demonstrated SGLT2is improved CVD outcomes with mildly reduced ejection fraction (EF) and HFpEF, and one study suggested possible benefit post-MI [36]. A newly published meta-analysis demonstrated the SGLT2i, dapagliflozin, reduced the risk of death from CV causes and hospital admissions for HF across a wide-range of EF [37]. Although these studies have not included an adequately identified Black cohort, these emerging data suggest that SGLT2is, with their proven BP-lowering effects and increasing benefit across a wide range of patients with HF regardless of EF along with renal and post-MI outcome benefits, may significantly add to the armamentarium of pharmacotherapeutic agents to reduce and potentially eliminate disparities in CVD outcomes in the non-Hispanic Black population. Evidence demonstrates that CVD incidence and mortality among racial and ethnic populations are among the highest in the U.S. Regardless, women and racial/ethnic populations continue to be underrepresented in CV clinical trials, relative to their disease burden and population percentage.

In addition to the lack of diverse participants in trials representing a moral and ethical issue, it also poses a scientific concern, as findings derived from trials that study homogenous groups of participants may not be generalizable to other genders, races, or ethnicities [38]. This unfortunate underrepresentation in trials may limit application of future therapies [39]. Recently, the FDA’s guidance, Collection of Race and Ethnicity Data in Clinical Trials, encourages clinical trial sponsors to compose a Race and Ethnicity Diversity Plan detailing enrollment and retention targets for a diverse study [40]. Furthermore, leaders of the Association of Black Cardiologists postulate that effective communication across key stakeholders is imperative for success in diverse trials [40]. Accordingly, a consortium of stakeholders convened to enhance HF therapeutic development (The Heart Failure Collaboratory) and to enhance recruitment strategies for patients from diverse and historically underrepresented groups [41]. Ultimately, diversifying trial enrollment is a feasible and efficient method to improve the generalizability and translation of trial knowledge to clinical practice.

## 7. Conclusions

In the final analysis, SGLTis will be beneficial in addressing HTN and associated CV risk in racial/ethnic populations, especially in non-Hispanic Black adults. CVD and CKD demonstrate benefits, including in HFrEF or HFpEF. SGLT2is lower clinic and out-of-office BP, attributed to natriuresis and osmotic diuresis, and additional mechanisms linking SGLT2is and neurohormonal activity are likely through the sympathetic nervous system. Therefore, SGLT2is are attractive choices for glycemic control, weight reduction, and BP-lowering with HTN, and with and without T2DM. Given the persistent and unacceptable disparities in CVD morbidity and mortality across sexes, race/ethnicities, and geographical regions in the U.S., it is critical to implement efforts to increase screening, access to healthcare, and a greater representation of diverse populations in clinical trials.
